# AVE0991, a nonpeptide analogue of Ang-(1-7), attenuates aging-related neuroinflammation

**DOI:** 10.18632/aging.101419

**Published:** 2018-04-17

**Authors:** Teng Jiang, Liu-Jun Xue, Yang Yang, Qing-Guang Wang, Xiao Xue, Zhou Ou, Qing Gao, Jian-Quan Shi, Liang Wu, Ying-Dong Zhang

**Affiliations:** 1 Department of Neurology, Nanjing First Hospital, Nanjing Medical University, Nanjing, PR China; 2 Department of Neurology, Huai'an First People's Hospital, Nanjing Medical University, Huai'an, PR China

**Keywords:** renin-angiotensin system, angiotensin-(1-7), AVE0991, neuroinflammation, microglia

## Abstract

During the aging process, chronic neuroinflammation induced by microglia is detrimental for the brain and contributes to the etiology of several aging-related neurodegenerative diseases such as Alzheimer’s disease and Parkinson’s disease. As a newly identified axis of renin-angiotensin system, ACE2/Ang-(1-7)/MAS1 axis plays a crucial role in modulating inflammatory responses under various pathological conditions. However, its relationship with aging-related neuroinflammation is less studied so far. In this study, by using SAMP8 mice, an animal model of accelerated aging, we revealed that the neuroinflammation in the aged brain might be attributed to a decreased level of Ang-(1-7). More importantly, we provided evidence that AVE0991, a nonpeptide analogue of Ang-(1-7), attenuated the aging-related neuroinflammation via suppression of microglial-mediated inflammatory response through a MAS1 receptor-dependent manner. Meanwhile, this protective effect might be ascribed to the M2 activation of microglia induced by AVE0991. Taken together, these findings reveal the association of Ang-(1-7) with the inflammatory response in the aged brain and uncover the potential of its nonpeptide analogue AVE0991 in attenuation of aging-related neuroinflammation.

## Introduction

In the process of aging, human brain displays a gradually enhanced inflammatory status called ‘inflamm-aging’ [1]. Although this status is crucial for the brain to remove senescent cells and extrinsic pathogenic substances, recent evidence has proven that chronic neuroinflammation is detrimental for the brain and may contribute to the etiology of various aging-related neurodegenerative diseases such as Alzheimer’s disease (AD) and Parkinson’s disease (PD) [2-4]. This notion was supported by previous findings that anti-inflammatory therapies could halt the progression of AD and PD in animal models [5,6].

Microglia are the major type of immune cells in the brain [7]. During the aging process, microglia can be activated and contribute to the neuroinflammation via release of pro-inflammatory cytokines [8,9]. This inflammatory response may subsequently lead to the injury or even death of neurons and synapses [10,11]. Therefore, microglia may represent a principal target for restricting the excessive neuroinflammation under disease status.

The renin-angiotensin system (RAS) has been accepted as an important modulator in the circulation system for maintaining the homeostasis of sodium and water [12]. In addition to the circulation system, cumulative evidence indicated that RAS also exists in colon, lungs, liver and brain, and exerts important physiological functions in these organs and tissues [13,14]. As a newly identified heptapeptide of RAS, angiotensin-(1-7) (Ang-(1-7)), along with angiotensin converting enzyme (ACE) 2 and MAS1 receptor, constitutes ACE2/Ang-(1-7)/MAS1 axis [15]. Emerging evidence revealed a crucial role of this axis in regulating inflammatory responses under various pathological conditions such as colitis, lung fibrosis, hepatic steatosis and ischemic stroke [16-19]. However, the relationship of brain ACE2/Ang-(1-7)/MAS1 axis with aging-related neuroinflammation is less studied thus far.

In this study, by using senescence-accelerated mouse prone 8 (SAMP8) mice, an animal model of accelerated aging [20,21], we revealed that the neuroinflammation in the aged brain might be attributed to a decreased level of Ang-(1-7). More importantly, we provided evidence that AVE0991, a nonpeptide analogue of Ang-(1-7), attenuated the aging-related neuroinflammation via suppression of microglial-mediated inflammatory response through a MAS1 receptor-dependent manner. Meanwhile, this protective effect might be ascribed to the M2 activation of microglia induced by AVE0991. Taken together, these findings reveal the association of Ang-(1-7) with the inflammatory response in the aged brain and uncover the potential of its nonpeptide analogue AVE0991 in attenuation of aging-related neuroinflammation.

## RESULTS

### Inflammatory markers are increased in the brains of SAMP8 mice during the aging process

**Figure 1:**
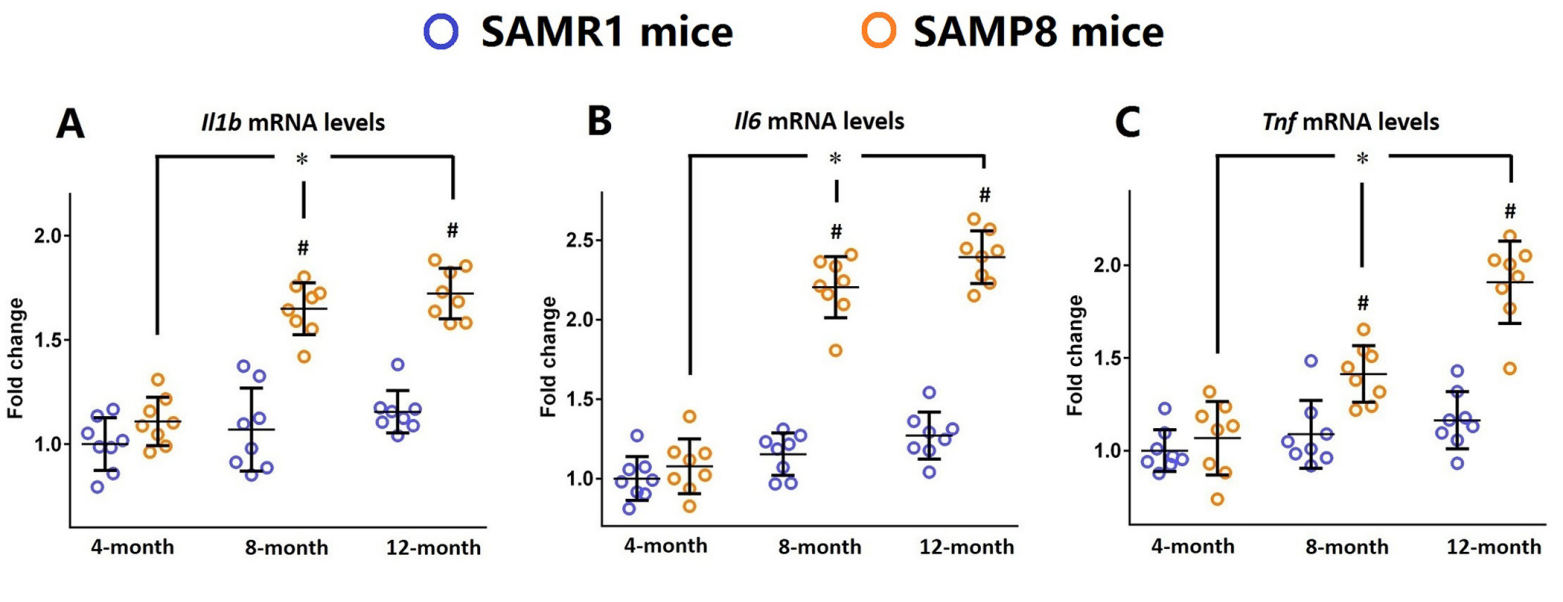
**Inflammatory markers are increased in the brains of SAMP8 mice during the aging process.** (**A**) The dynamic changes of *Il1b* mRNA levels in the brains of 4-, 8-, and 12-month-old SAMP8 mice as well as their age-matched SAMR1 control mice were investigated by qRT-PCR. (**B**) The dynamic changes of *Il6* mRNA levels in the brains of 4-, 8-, and 12-month-old SAMP8 mice as well as their age-matched SAMR1 control mice were investigated by qRT-PCR. (**C**) The dynamic changes of *Tnf* mRNA levels in the brains of 4-, 8-, and 12-month-old SAMP8 mice as well as their age-matched SAMR1 control mice were investigated by qRT-PCR. *Gapdh* was used as an internal control. All data were analyzed by one-way ANOVA followed by Tukey’s post hoc test and were expressed as a fold change relative to 4-month-old SAMR1 control mice. Columns represent mean ± SD (n=8 per group). *P<0.05. #*P*<0.05 versus age-matched SAMR1 control mice.

First, the dynamic changes of inflammatory markers including IL-1β, IL-6, and TNF-α in the brains of 4-, 8-, and 12-month-old SAMP8 mice as well as their age-matched controls were investigated by qRT-PCR. As shown in Figure 1, the mRNA levels of *Il1b*, *Il6* and *Tnf* were significantly increased in the brains of SAMP8 mice during the aging process (n=8, *P*<0.05). Meanwhile, a significant higher expression of *Il1b*, *Il6* and *Tnf* was observed in the brains of 8- and 12-month-old SAMP8 mice when compared with their age-matched controls (Figure 1, n=8, *P*<0.05). These findings indicated an increased neuroinflammatory response in the aged brain.

### Ang-(1-7) levels are decreased in the brains of SAMP8 mice during the aging process

**Figure 2:**
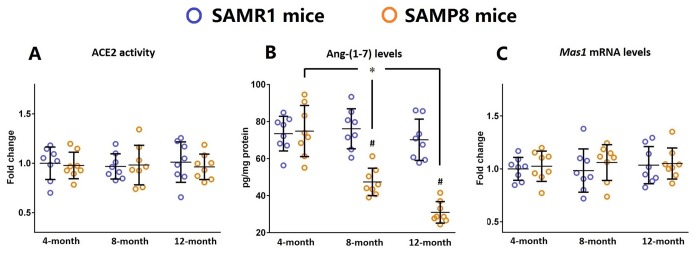
**Ang-(1-7) levels are decreased in the brains of SAMP8 mice during the aging process.** (**A**) The activity of ACE2 in the brains of 4-, 8-, and 12-month-old SAMP8 mice as well as their age-matched SAMR1 control mice were assessed using a specific detection kit. (**B**) The Ang-(1-7) levels in the brains of 4-, 8-, and 12-month-old SAMP8 mice as well as their age-matched SAMR1 control mice were detected by ELISA. (**C**) The expression of *Mas1* mRNA levels in the brains of 4-, 8-, and 12-month-old SAMP8 mice as well as their age-matched SAMR1 control mice were assessed by qRT-PCR. *Gapdh* was used as an internal control. Data from panel **B** and **C** were expressed as a fold change relative to 4-month-old SAMR1 control mice. All data were analyzed by one-way ANOVA followed by Tukey’s post hoc test. Columns represent mean ± SD (n=8 per group). *P<0.05. #*P*<0.05 versus age-matched SAMR1 control mice.

Next, we determined whether this increased neuroinflammatory response was related to any alterations in the components of brain ACE2/Ang-(1-7)/MAS1 axis. As shown in Figure 2, in the brains of SAMP8 mice, the levels of Ang-(1-7) were markedly decreased during the aging process (n=8, *P*<0.05). Meanwhile, the Ang-(1-7) levels in the brains of 8- and 12-month-old SAMP8 mice were significantly lower when compared with those of their age-matched controls (n=8, *P*<0.05). It should be noted that the activity of ACE2 and the mRNA level of *Mas1* remained stable in the brains of SAMP8 mice during the aging process. These findings implied that the increased neuroinflammatory response might be attributed to the decreased Ang-(1-7) levels in the aged brain.

### AVE0991 attenuates neuroinflammation in the brains of SAMP8 mice

Based on the above findings, we then hypothesized that restoration of Ang-(1-7) levels might attenuate neuroinflammation in the aged brain. Since Ang-(1-7) will be rapidly inactivated and degraded by several proteases and thus has a relatively short duration of biological effect *in vivo*, we employed AVE0991, a nonpeptide analogue of Ang-(1-7) [22], in the subsequent experiments.

**Figure 3:**
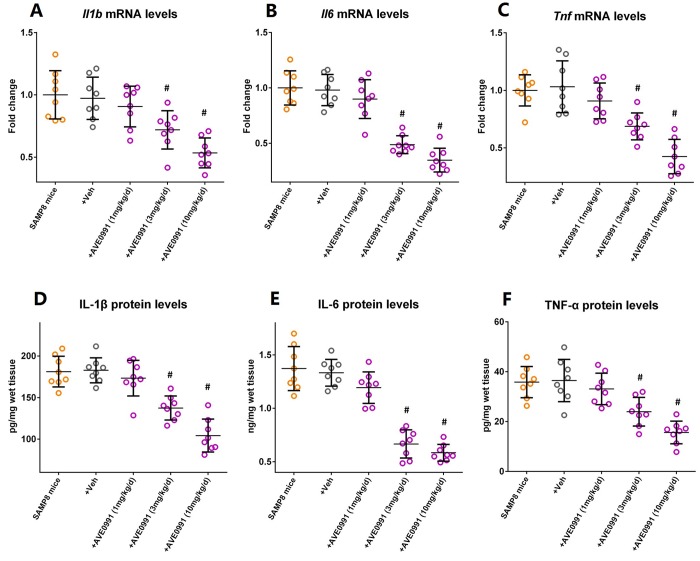
**AVE0991 attenuates neuroinflammation in the brains of SAMP8 mice.** Eight-month-old SAMP8 mice were injected intraperitoneally with vehicle or AVE0991 (1, 3 or 10 mg/kg/day) for 30 days. Afterwards, mice were sacrificed for analysis. (**A**) The mRNA levels of *Il1b* in the brains were investigated by qRT-PCR. (**B**) The mRNA levels of *Il6* in the brains were investigated by qRT-PCR. (**C**) The mRNA levels of *Tnf* in the brains were investigated by qRT-PCR. (**D**) The protein levels of IL-1β in the brains were investigated by ELISA. (**E**) The protein levels of IL-6 in the brains were investigated by ELISA. (**F**) The protein levels of TNF-α in the brains were investigated by ELISA. In panel **A**-**C,** *Gapdh* was used as an internal control, and data were expressed as a fold change relative to non-treated SAMP8 mice. All data were analyzed by one-way ANOVA followed by Tukey’s post hoc test. Columns represent mean ± SD (n=8 per group). #*P*<0.05 versus vehicle-treated SAMP8 mice.

As illustrated by Supplementary Figure S1, eight-month-old SAMP8 mice were injected intraperitoneally with vehicle or AVE0991 (1, 3 or 10 mg/kg/day) once a day for 30 consecutive days. AVE0991 injection (3 or 10 mg/kg/day) significantly decreased the mRNA levels of *Il1b*, *Il6* and *Tnf* in the brains of SAMP8 mice (Figure 3A-C, n=8, *P*<0.05). In addition, a corresponding decrease in protein levels of IL-1β, IL-6 and TNF-α in the brains of SAMP8 mice was observed following AVE0991 (3 or 10 mg/kg/day) injection (Figure 3D-F, n=8, *P*<0.05). It should be noted that AVE0991 (1, 3 or 10 mg/kg/day) had no significant effect on mRNA levels of *Il1b*, *Il6* and *Tnf* in the brains of 8-month-old SAMR1 control mice (Preliminary experiments, Supplementary Figure S2, n=5). These findings indicated that AVE0991 attenuated neuroinflammation in the aged brain.

### AVE0991 suppresses microglial-mediated inflammatory response through a MAS1 receptor-dependent manner

**Figure 4:**
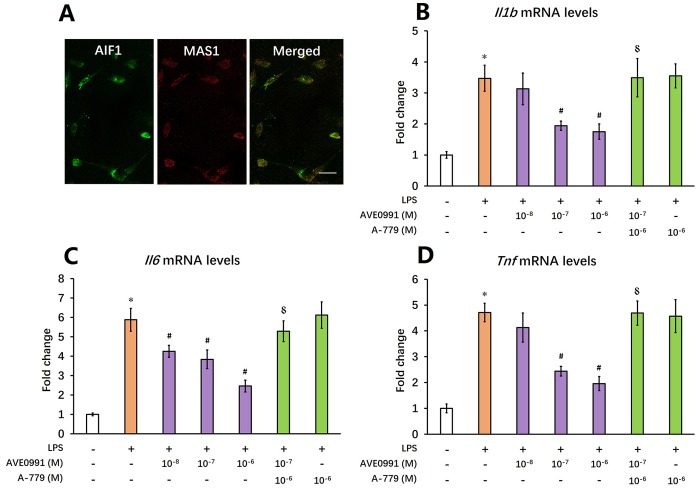
**AVE0991 suppresses microglial-mediated inflammatory response through a MAS1 receptor-dependent manner.** Primary microglia were directly isolated from the brain of 8-month-old SAMP8 mice. (**A**) The localization of MAS1 receptor on microglia was confirmed by double immunofluorescence staining. Scale bar=15 μm. Next, microglia were treated for 4 h with 100 ng/ml LPS with or without 4 h pre-incubation with AVE0991 (1×10^-8^, 1×10^-7^ or 1×10^-6^ M) in the presence or absence of A-779 (1×10^-6^ M) and were harvested and lysed for analysis. (**B**) The mRNA levels of *Il6* were investigated by qRT-PCR. (**C**) The mRNA levels of *Ilb* were investigated by qRT-PCR. (**D**) The mRNA levels of *Tnf* were investigated by qRT-PCR. In panel **B**-**D,** *Gapdh* was used as an internal control, and data were expressed as a fold change relative to non-treated microglia. All data were analyzed by one-way ANOVA followed by Tukey’s post hoc test. Columns represent mean ± SD (n=4-6). *P<0.05 versus non-treated microglia. #*P*<0.05 versus microglia incubated with LPS alone. ⸹*P*<0.05 versus microglia treated with LPS+AVE0991 (1×10^-7^M).

Afterwards, we tried to elucidate the underlying mechanisms by which AVE0991 attenuated neuroinflammation. As MAS1 receptor was reported to be expressed by microglia whilst activation of microglia contributed to the neuroinflammatory response during the aging process [8,23], we then speculated that AVE0991 attenuated neuroinflammation by suppressing microglial-mediated inflammatory response. To test this hypothesis, primary microglia were isolated from the brain of 8-month-old SAMP8 mice. Double immunofluorescence staining indicated a good co-localization of MAS1 with a microglial marker AIF1, confirming that MAS1 receptor is expressed by microglia (Figure 4A). Next, microglia were treated for 4 h with 100 ng/ml LPS with or without 4 h pre-incubation with AVE0991 (1×10^-8^, 1×10^-7^ or 1×10^-6^ M) in the presence or absence of A-779 (1×10^-6^ M). LPS incubation induced an inflammatory response in microglia, since the mRNA levels of *Il1b*, *Il6* and *Tnf* were significantly increased (Figure 4B-D, *P*<0.05). Pre-incubation with AVE0991 (1×10^-7^ or 1×10^-6^ M) attenuated the LPS-induced increase in *Il1b* and *Tnf* mRNA levels (Figure 4B-D, *P*<0.05). Meanwhile, the increase in *Il6* mRNA levels was markedly inhibited by AVE0991 (1×10^-8^, 1×10^-7^ or 1×10^-6^ M). It is worthy to note that the effects of AVE0991 (1×10^-7^ M) on the mRNA levels of *Il1b*, *Il6* and *Tnf* were fully abolished by A-779 (1×10^-6^ M), an antagonist of MAS1 receptor (Figure 4B-D, *P*<0.05). In addition, A-779 (1×10^-6^ M) alone did not significantly influence the expression of these inflammatory markers (Figure 4B-D).

### AVE0991 promotes M2 activation of microglia

**Figure 5:**
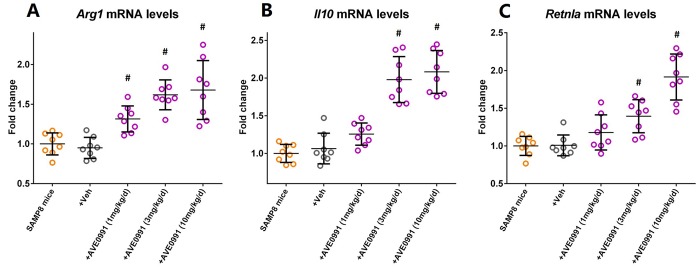
**AVE0991 elevates microglial M2 activation makers in the brains of SAMP8 mice.** Eight-month-old SAMP8 mice were injected intraperitoneally with vehicle or AVE0991 (1, 3 or 10 mg/kg/day) for 30 days. Afterwards, mice were sacrificed for analysis. (**A**) The mRNA levels of *Arg1* in the brains were investigated by qRT-PCR. (**B**) The mRNA levels of *Il10* in the brains were investigated by qRT-PCR. (**C**) The mRNA levels of *Retnla* in the brains were investigated by qRT-PCR. *Gapdh* was used as an internal control, and data were expressed as a fold change relative to non-treated SAMP8 mice. All data were analyzed by one-way ANOVA followed by Tukey’s post hoc test. Columns represent mean ± SD (n=8 per group). #*P*<0.05 versus vehicle-treated SAMP8 mice.

**Figure 6:**
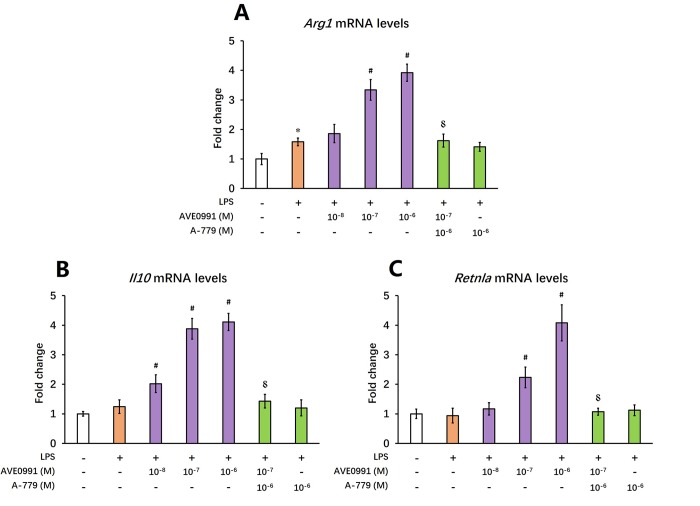
**AVE0991 elevates M2 activation makers in primary microglia.** Primary microglia were directly isolated from the brain of 8-month-old SAMP8 mice. They were treated for 4 h with 100 ng/ml LPS with or without 4 h pre-incubation with AVE0991 (1×10^-8^, 1×10^-7^ or 1×10^-6^ M) in the presence or absence of A-779 (1×10^-6^ M) and were harvested and lysed for analysis. (**A**) The mRNA levels of *Arg1* were investigated by qRT-PCR. (**B**) The mRNA levels of *Il10* were investigated by qRT-PCR. (**C**) The mRNA levels of *Retnla* were investigated by qRT-PCR. *Gapdh* was used as an internal control, and data were expressed as a fold change relative to non-treated microglia. All data were analyzed by one-way ANOVA followed by Tukey’s post hoc test. Columns represent mean ± SD (n=4-6). *P<0.05 versus non-treated microglia. #*P*<0.05 versus microglia incubated with LPS alone. ⸹*P*<0.05 versus microglia treated with LPS+AVE0991 (1×10^-7^M).

As reduced inflammatory response represents a major characteristic of M2 activation of microglia [24], we then hypothesized that AVE0991 suppressed microglial-mediated inflammatory response by switching microglia toward the M2 activation. To test this hypothesis, we measured the mRNA levels of M2 activation makers including *Arg1*, *Il10*, and *Retnla* in the brains of SAMP8 mice following AVE0991 treatment. Injection of AVE0991 (1, 3 or 10 mg/kg/day for 30 consecutive days) markedly increased the mRNA levels of *Arg1* in the brains of SAMP8 mice (Figure 5A, *P*<0.05). Meanwhile, the mRNA levels of *Il10* and *Retnla* in the brains of SAMP8 mice were significantly elevated following AVE0991 injection (3 or 10 mg/kg/day for 30 consecutive days, Figure 5B and C, *P*<0.05). To further validate this finding, microglia isolated from 8-month-old SAMP8 mice were treated for 4 h with 100 ng/ml LPS with or without 4 h pre-incubation with AVE0991 (1×10^-8^, 1×10^-7^ or 1×10^-6^ M) in the presence or absence of A-779 (1×10^-6^ M). As shown in Figure 6A and C, pre-incubation with AVE0991 (1×10^-7^ or 1×10^-6^ M) significantly increased the mRNA levels of *Arg1* and *Retnla* (*P*<0.05). Meanwhile, the mRNA levels of *Il10* were markedly elevated by AVE0991 (1×10^-8^, 1×10^-7^ or 1×10^-6^ M, Figure 6B, *P*<0.05). It should be noted that the effects of AVE0991 (1×10^-7^ M) on *Arg1*, *Il10*, and *Retnla* mRNA levels were fully reversed by A-779 (1×10^-6^ M, Figure 6, *P*<0.05). Additionally, A-779 (1×10^-6^ M) itself did not markedly affect the expression of these M2 activation markers (Figure 6).

## DISCUSSION

First, in this study, we showed a progressively enhanced inflammatory response in the aged brain, since the expression of inflammatory markers including IL-1β, IL-6, and TNF-α was significantly increased in SAMP8 mice brain during the aging process. These results were compatible with previous findings from our group and others that SAMP8 mice exhibited a chronic neuroinflammatory status [25,26]. Meanwhile, we demonstrated that the levels of Ang-(1-7) was significantly reduced in the aged brain, and these findings were in accordance with our previous observations in SAMP8 mice [27].

As a recently identified bioactive peptide of RAS, Ang-(1-7) is produced mainly from angiotensin II by ACE2 and binds to MAS1 receptor for its signaling and biologic functions [28]. Emerging evidence suggested that Ang-(1-7) could inhibit inflammatory responses under various pathological conditions. In an experimental model of colitis, daily Ang-(1-7) treatment significantly ameliorated inflammation induced by dextran sulfate sodium [16]. Meanwhile, the anti-inflammatory effects of Ang-(1-7) were also observed in a mouse model of lung fibrosis induced by bleomycin [17]. Moreover, treatment with an oral formulation of Ang-(1-7) suppressed the expression of inflammatory markers and improved hepatic functions in a mouse model of hepatic steatosis caused by a high-fat diet [18]. In addition, in a rat model of ischemic stroke, Ang-(1-7) infusion exerted neuroprotective effects via inhibition of NF-κB-mediated inflammatory response [19]. In view of the anti-inflammatory actions of Ang-(1-7), we speculated that the enhanced inflammatory response in the brain during the aging process might attributed to the reduction of Ang-(1-7) levels, and restoration of Ang-(1-7) levels might attenuate this aging-related neuroinflammation.

However, it should be noted that Ang-(1-7) has a relatively short duration of biological effect *in vivo* since it can be rapidly inactivated and degraded by several proteases such as ACE and neutral endopeptidase [29-31]. To avoid theses disadvantages of Ang-(1-7), we then employed its nonpeptide analogue AVE0991 in the subsequent experiments to further verify our hypothesis [22]. As expected, AVE0991 treatment ameliorated the aging-related neuroinflammation in SAMP8 mice, since the expression of inflammatory markers was markedly reduced. To our knowledge, this is the first study to reveal the anti-inflammatory property of AVE0991 in an animal model of accelerated aging.

Afterwards, we tried to elucidate the underlying mechanisms by which AVE0991 attenuated neuroinflammation. As revealed by our double immunofluorescence staining, MAS1 receptor was expressed by microglia, and this observation was supported by an earlier study from Liu and colleagues showing that MAS1 receptor was stably expressed by human microglia [23]. As the main immune cells in the brain, microglia was activated during the aging process and thus contributed to the chronic neuroinflammation via release of pro-inflammatory cytokines [8,9]. In this study, we revealed that AVE0991 treatment suppressed the LPS-induced expression of inflammatory markers in primary microglia isolated from SAMP8 mice, and this effect can be completely abolished by an antagonist of MAS1 receptor A-779. This observation indicated that AVE0991 attenuated neuroinflammation by suppressing microglial-mediated inflammatory response via a MAS1 receptor-dependent manner. More interestingly, an increased level of microglial M2 activation markers including *Arg1*, *Retnla* and *Il10* was observed following AVE0991 treatment. In contrast to M1 activation, M2 activation of microglia is featured by a suppressed inflammatory response as well as an enhanced phagocytic activity [24]. Therefore, it seemed that the anti-inflammatory actions of AVE0991 were achieved by switching microglia toward the M2 activation in this scenario.

It is noteworthy that the current study also has some limitations. For example, SAMP8 mouse used in this study is an animal model of accelerated aging. Its pathophysiological process and neuropathology are not equal to those under normal aging conditions [20,21]. Therefore, the current findings in this study need to be further confirmed using normal aging mice in future.

**Figure 7:**
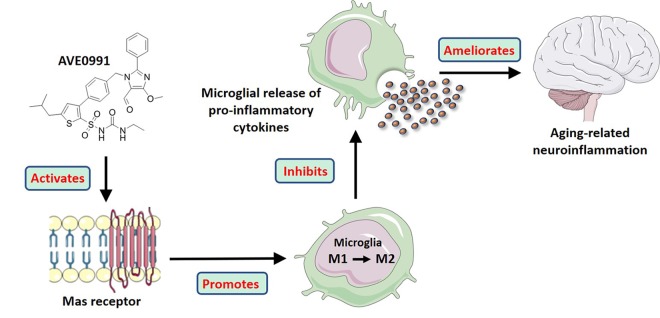
**A model illustrating the effects of AVE0991 on aging-related neuroinflammation and its potential mechanisms.**

Summarily, in this study, we revealed that the neuroinflammation in the aged brain might be attributed to a decreased level of Ang-(1-7). More importantly, we provided evidence that AVE0991, a nonpeptide analogue of Ang-(1-7), attenuated the aging-related neuroinflammation via suppression of microglial-mediated inflammatory response through a MAS1 receptor-dependent manner. Meanwhile, this protective effect might be ascribed to the M2 activation of microglia induced by AVE0991 (Summarized in Figure 7). Taken together, these findings reveal the association of Ang-(1-7) with the inflammatory response in the aged brain and uncover the potential of its nonpeptide analogue AVE0991 in attenuation of aging-related neuroinflammation.

## METHODS

### Reagents

AVE0991, a nonpeptide agonist of MAS1 receptor [22], was purchased from Medchem Express Inc. A-779, a selective antagonist of MAS1 receptor, was purchased from Abcam Inc.

### Animals and treatments

To avoid the interference of estrogen on neuroinflammation, only male animals were used in this study [32-34]. Four-, eight- and twelve-month-old male SAMP8 mice and their age-matched senescence-accelerated mouse resistant 1 (SAMR1) control mice were purchased from the Institute of Zoology, Chinese Academy of Sciences. They were maintained in individually ventilated cages in a standard animal room with a 12 h light/dark cycle and given free access to food and water as described [35]. This study was reported in accordance with the ARRIVE guideline, and the procedures in this study involving animals were in accordance with the ethical standards of Nanjing First Hospital.

Eight-month-old male SAMP8 mice were randomly allocated to five groups using a random number table generated by SPSS software, and received treatments as shown in Supplementary Figure S1. During treatment, we monitored the general health of mice and did not observe any adverse effects or significant changes in their systolic blood pressure, body weights or food intakes (data not shown).

### Primary microglia cultures and treatments

Microglia were isolated from the brains of 8-month-old SAMP8 mice using a protocol described by Bliederhaeuser and colleagues with our minor modification [36-38]. Briefly, mice were deeply anesthetized using an overdose of chloral hydrate and perfused with ice-cold Ringers solution. Brains were fragmented with a scalpel and incubated with an enzymatic solution containing papain. The papain solution was quenched with 20% (FBS) in Hank’s balanced salt solution (HBSS) and centrifuged for 4 min at 200g. The pellet was resuspended in 2 mL of 0.5 mg/mL DNase in HBSS and incubated for 5 min. After being filtered through a 70-μm cell strainer and centrifuged at 200g for 4 min, the resulting pellet was then resuspended in 20% isotonic Percoll® (Sigma-Aldrich, Inc.) in HBSS. Pure HBSS was carefully laid on the top of the “percoll” layer and centrifugation was performed at 200g for 20 min. The interphase layer containing myelin and cell debris was discarded, and the pellet containing the mixed glial cell population was washed once with HBSS and suspended in Dulbecco’s modified Eagle’s medium supplemented with 10% FBS, 1%P/S, 1×pyruvate, 1×GlutaMAX™ (Thermo Fisher Scientific, Inc.) as well as 5 ng/mL of murine recombinant granulocyte and macrophage colony stimulating factor (GM-CSF). The cell suspension was plated on a 15 cm^2^ plate coated with poly-l-lysine and maintained in culture at 37°C, 5% CO_2_. The medium was replaced every 3 days until the cells reached confluency (after approximately 14-21 days *in vitro* (DIV)). After the glial layer becomes confluent, microglia form a nonadherent, floating cell layer. After collecting the floating layer, microglia were incubated for 3 days without GM-CSF before analysis or receiving treatments (17-24 DIV). The purity of microglia was confirmed by immunocytochemistry.

Microglia were treated for 4 h with 100 ng/ml LPS with or without 4 h pre-incubation with AVE0991 (1x10^-8^, 1x10^-7^ or 1x10^-6^ M) in the presence or absence of A-779 (1x10^-6^ M). During these treatments, cell viability was not significantly affected (data not shown). Afterwards, cells were collected for the subsequent analyses.

### Double immunofluorescence assay

Double immunofluorescence assay was performed as described [39,40]. Microglia were fixed with 75% methanol in water for 10 min at -20 °C, washed with 1 mL phosphate buffer saline (PBS), and permeabilized with 0.5 mL 0.2% Triton X-100 in PBS for 15 min. Prior to the addition of antibody, cells were blocked in a blocking buffer containing PBS, 0.1% Triton X-100, and 4% bovine serum albumin (BSA) overnight. A mouse monoclonal antibody against MAS1 (1:50, Santa Cruz Biotechnology, Inc. USA) and a rabbit monoclonal antibody against AIF1 (1:100, Abcam Inc. USA) were added to the solution and incubated with the cells overnight. After washing thrice with PBS (pH 7.5) for 10 min, the cells were incubated with a TRITC-conjugated anti-mouse IgG secondary antibody (Zhongshan Goldenbridge Biotechnology, China) and a FITC-conjugated anti-rabbit IgG secondary antibody (Zhongshan Goldenbridge Biotechnology, China) in PBS with 0.1% Triton X-100 and 1% BSA for 1 h at room temperature and were viewed using a Nikon fluorescence microscope.

### Brain tissue preparation

After indicated treatment, mice were killed under deep anesthesia through an overdose of chloral hydrate and were perfused transcardially with 0.9% saline (pH 7.4). Brains were then removed and stored in liquid nitrogen until use.

### Quantitative Reverse Transcription PCR (qRT-PCR)

Total RNA in the whole brains or isolated mouse primary microglia was extracted by Trizol reagent as described [37,41]. Equal amounts of total RNA were reverse transcribed under standard conditions using the PrimeScript™ RT Master Mix (Takara Bio, Inc.). After that, qRT-PCR reactions were performed with SYBR Green Premix Ex Taq (Takara Bio, Inc.) and specific primers (See Supplementary Table S1).

### Measurement of brain ACE2 activity

ACE2 activity in mice brain was measured by a SensoLyte 390 ACE2 activity assay kit (AnaSpec) with Mc-Ala/Dnp fluorescence resonance energy transfer peptides as described [27]. The fluorescence of Mc-Ala was monitored at excitation/emission 330/390 nm. Reactions were performed in duplicate in 96-well, clear, flat-bottom polystyrene microplates at a final volume of 100 μL. Specificity was confirmed using DX600, a specific ACE2 inhibitor (AnaSpec).

### Enzyme-linked immunosorbent assay (ELISA)

The concentrations of Ang-(1-7) and inflammatory markers including IL-1β, IL-6 and TNF-α in mice brain were measured by specific ELISA kits (R&D Systems, Inc.) as described [42,43]. The alteration in absorbance in every well at 450 nm was detected with a microplate reader.

### Statistical analysis

Data were analyzed using GraphPad Prism 6 (GraphPad Software, Inc.). Difference between mean values was determined by one-way analysis of variance (ANOVA) followed by Tukey’s post hoc test. Data are expressed as mean ± SD. *P*<0.05 was considered statistically significant.

## Supplementary Material

Supplementary File
