# Supplementary material for: AVE0991, a nonpeptide analogue of Ang-(1-7), attenuates aging-related neuroinflammation
Source: Aging (Albany NY). 2018 Apr 17;10(4):645–57. doi: 10.18632/aging.101419 (PMC5940107; doi:10.18632/aging.101419)
Supplement: Supplementary File [file aging-10-101419-s001.pdf]

SUPPLEMENTARY MATERIAL

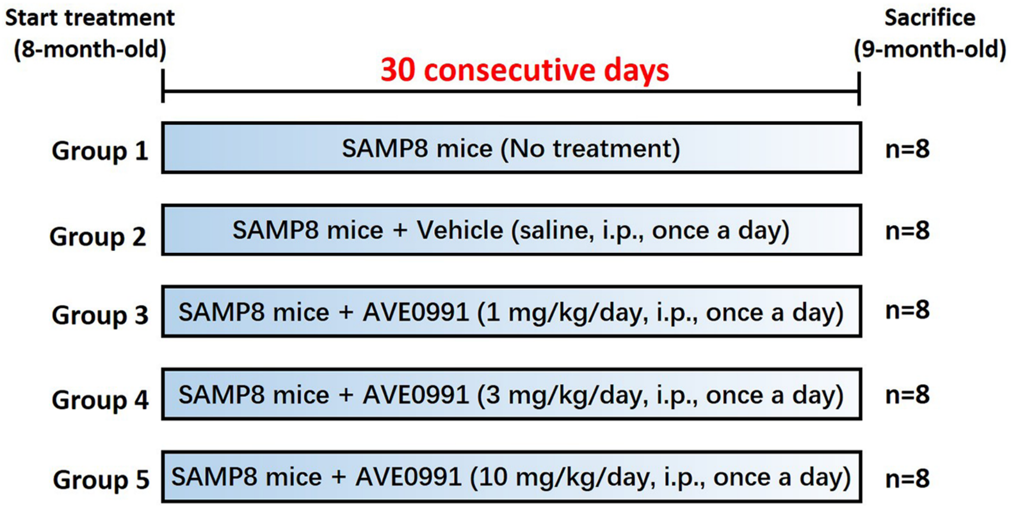

Supplementary Figure 1. Scheme of the animal experimental protocol. Eight-month-old SAMP8 mice were randomly allocated to five groups using a random number table generated by SPSS software (n=8 per group). They were injected intraperitoneally with vehicle (saline) or AVE0991 (1 mg/kg/day, 3 mg/kg/day or 10 mg/kg/day) once a day for 30 consecutive days.

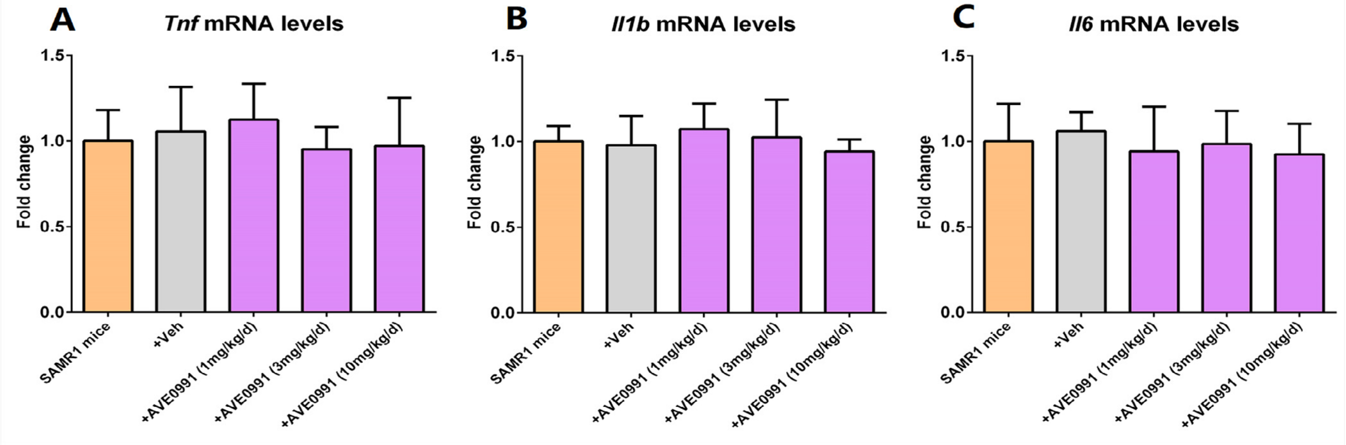

Supplementary Figure 2. AVE0991 has no significant effect on inflammatory markers in the brains of SAMR1 control mice. In our preliminary experiments, eight-month-old SAMR1 mice were injected intraperitoneally with vehicle or AVE0991 (1, 3 or 10 mg/kg/day) for 30 days. Afterwards, mice were sacrificed for analysis. (**A**) The mRNA levels of *Tnf* in the brains were investigated by qRT-PCR. (**B**) The mRNA levels of *Il1b* in the brains were investigated by qRT-PCR. (**C**) The mRNA levels of *Il6* in the brains were investigated by qRT-PCR. *Gapdh* was used as an internal control, and data were expressed as a fold change relative to non-treated SAMR1 mice. All data were analyzed by one-way ANOVA followed by Tukey's post hoc test. Columns represent mean ± SD (n=5 per group).

Supplementary Table 1. Oligonucleotide sequences.

Primer	Sequence (5'-3')	GenBank accession number*
Arg1 forward	CTCCAAGCCAAAGTCCTTAGAG	NM_007482.3
Arg1 reverse	AGGAGCTGTCATTAGGGACATC	
Gapdh forward	CAACAGCAACTCCCACTCTTC	NM_001289726.1
Gapdh reverse	GGTCCAGGGTTTCTTACTCCTT	
Il1b forward	GAAGAGCCCATCCTCTGTGA	NM_008361.3
Il1b reverse	TTCATCTCGGAGCCTGTAGTG	
Il6 forward	ACAAAGCCAGAGTCCTTCAGAG	NM_031168.1
Il6 reverse	CATTGGAAATTGGGGTAGGA	
Il10 forward	AGGCGCTGTCATCGATTTCTC	NM_019467.2
Il10 reverse	TGCTCCACTGCCTTGCTCTTA	
Mas1 forward	CATCTAGGACTGGGCAGAGC	NM_008552.5
Mas1 reverse	AGTCAGGAGGTGGAGAGCAA	
Retnla forward	TTGCAACTGCCTGTGCTTAC	NM_020509.3
Retnla reverse	CAAGAAGCAGGGTAAATGGG	
Tnf forward	GTCTACTGAACTTCGGGGTGAT	NM_013693.3
Tnf reverse	ATGATCTGAGTGTGAGGGTCTG	

*The GenBank accession numbers were obtained from the NCBI.
